# Effect of EV71 Vaccination on Transmission Dynamics of Hand, Foot, and Mouth Disease and Its Epidemic Prevention Threshold

**DOI:** 10.3390/vaccines12101166

**Published:** 2024-10-12

**Authors:** Dashan Zheng, Lingzhi Shen, Wanqi Wen, Zitong Zhuang, Samantha E. Qian, Feng Ling, Ziping Miao, Rui Li, Stephen Edward McMillin, Sabel Bass, Jimin Sun, Hualiang Lin, Kun Liu

**Affiliations:** 1 Department of Epidemiology, School of Public Health, Sun Yat-sen University, Guangzhou 510080, China; zhengdsh5@mail2.sysu.edu.cn (D.Z.); linhualiang@mail.sysu.edu.cn (H.L.); 2 Zhejiang Provincial Center for Disease Control and Prevention, Hangzhou 310051, China; 3 College of Arts and Sciences, Saint Louis University, Saint Louis, MO 63108, USA; 4 Department of Epidemiology, Ministry of Education Key Lab of Hazard Assessment and Control in Special Operational Environment, School of Public Health, Air Force Medical University, 169 Changle West Road, Xi’an 710032, China; 5 School of Social Work, Saint Louis University, Saint Louis, MO 63103, USA; 6 Department of Epidemiology and Biostatistics, College for Public Health and Social Justice, Saint Louis University, Saint Louis, MO 63104, USA

**Keywords:** hand, foot and mouth disease, transmissibility, Enterovirus A71 (EV71) vaccine, effective reproduction number

## Abstract

Objective: To investigate the effect of Enterovirus A71 (EV71) vaccination on the transmissibility of different enterovirus serotypes of hand, foot, and mouth disease (HFMD) in Zhejiang, China. Methods: Daily surveillance data of HFMD and EV71 vaccination from August 2016 to December 2019 were collected. Epidemic periods for each HFMD type were defined, and the time-varying effective reproduction number (R_t_) was estimated, which could provide more direct evidence of disease epidemics than case number. General additive models (GAMs) were employed to analyze associations between EV71 vaccination quantity and rate and HFMD transmissibility. The epidemic prevention threshold, represented by required vaccination numbers and rates, was also estimated. Results: Vaccinating every 100,000 children ≤ 5 years could lead to a decrease in the *R_t_* of EV71-associated HFMD by 14.44% (95%CI: 6.76%, 21.42%). Additionally, a positive correlation was observed between vaccinations among children ≤ 5 years old (per 100,000) and the increased transmissibility of other HFMD types (caused by enteroviruses other than EV71 and CA16) at 1.82% (95%CI: 0.80%, 2.84%). It was estimated that an additional 362,381 vaccinations, corresponding to increased vaccine coverage to 54.51% among children ≤ 5 years could effectively prevent EV71 epidemics in Zhejiang. Conclusions: Our findings highlight the importance of enhancing EV71 vaccine coverage for controlling the epidemic of EV71-HFMD and assisting government officials in developing strategies to prevent HFMD.

## 1. Introduction

Hand, foot, and mouth disease (HFMD) is an acute enteric viral infection and a significant public health concern [1]. Various enteroviruses, such as Enterovirus A71 (EV71), Coxsackievirus A16 (CA16), and other intestinal viruses, can cause a broad spectrum of diseases, particularly HFMD [2,3]. Among them, EV71 is identified as the primary pathogen linked to severe cases and fatalities [4]. In the last few decades, the Asian-Pacific region, including China, Malaysia, and Japan, experienced severe epidemics of HFMD [5]. Thus, China included HFMD as a notifiable infectious disease in 2008 and developed the inactivated EV71 vaccine [6].

The vaccine was licensed for administration after proving effectiveness in phases III clinical trials among health children (6 to 71 months of age) with 97.4% vaccine efficacy in intention-to-treat analysis and 97.3% in per-protocol analysis [7]. Furthermore, existing real-world studies have primarily evaluated the efficacy of EV71 vaccination based on the number of cases. For instance, one population surveillance study in Chengdu, China, assessed that 60% of EV71-associated HFMD cases could be prevented by EV71 vaccination [8]. Another hospital-based study also reported vaccine effectiveness of 85.4% for those fully vaccinated and 63.1% for those partially vaccinated [9].

However, the number of cases, as an outcome measure, may not directly reveal the effects of vaccination on HFMD transmission dynamics, as it depends on the combined effect of transmissibility and the time to symptom appearance [10]. Alternatively, investigating how vaccines impact the transmissibility of HFMD could provide more direct evidence for preventing HFMD epidemics. Nevertheless, there is still a dearth of evidence concerning the relationship between vaccines and HFMD transmissibility [11]. Furthermore, existing research is limited to evaluating the effectiveness of the EV71 vaccine or the strength of its effects, without estimating the threshold for preventive vaccination.

To bridge these research gaps, we gathered surveillance data from Zhejiang Province and computed the time-varying effective reproduction number (*R_t_*) to represent HFMD transmissibility. Furthermore, we employed the general additive model (GAM) to analyze the associations of vaccination number and rate with *R_t_* and further estimated the required vaccination number or rates to prevent an EV71 epidemic.

## 2. Methods

### 2.1. Data Source and Description

Zhejiang Province (118° E–123° E, 27° N–32° N), situated on the southeast coast of China, boasts a humid atmosphere, a temperate climate, and a flourishing economy. With 11 cities and an area of 101,800 square kilometers, Zhejiang Province is one of the most populated provinces in China. Notably, it has also consistently ranked among the top three Chinese provinces in terms of HFMD incidence [12], with EV71 as its main serotype [13].

According to the Chinese Guidelines for the Diagnosis and Treatment of HFMD [14], the diagnostic criteria of HFMD is determined based on current epidemiological and clinical manifestations and virological investigations [14,15]. Specifically, a history of contact with patients of hand, foot, and mouth disease prior to the onset of symptoms, or the presence of hand, foot, and mouth disease outbreaks in local childcare facilities and surrounding communities, along with typical clinical manifestations such as fever and rash on the hands, feet, mouth, or buttocks, or atypical presentations such as encephalitis or meningitis, combined with microbiological or serological test results, can lead to a diagnosis. Subsequently, diagnosed cases that meet the above criteria must be reported to the National Notifiable Infectious Diseases Reporting Information System (NNIDRIS) immediately [16]. Considering the start of the vaccine (August 2016) and the COVID-19 pandemic in 2020, which significantly changed children’s lifestyles and habits, we collected HFMD cases in Zhejiang Province from August 2016 to December 2019 from NNIDRIS. The information included gender, age, occupation, location, and the date of incidence.

### 2.2. Estimation of EV71 Cases and Vaccine Coverage

We gathered etiological data from Zhejiang Province Center for Disease Control and Prevention (CDC). Samples from reported cases of HFMD were selected for laboratory analysis to differentiate EV71, CA16, and other enteroviruses (caused by enteroviruses other than EV71 and CA16), by using techniques such as PCR amplification of the VP1 gene region and subsequent sequencing to genotype the viral RNA [2,12]. Real-time reverse transcription polymerase chain reaction (RT-PCR) was employed for the laboratory analyses. Given that not all recorded HFMD cases underwent etiological detection, we utilized the following equation to estimate the daily counts of different serotypes of HFMD cases [17]:

Daily cases of EV71 (or CA16, other serotype) = Daily HFMD cases × Daily EV71 (or CA16, other serotype) positive rate from etiological detection.

According to the technical guidelines for the use of enterovirus 71 inactivated vaccine, each vaccine recipient needs to complete two doses of injections 28 days apart; the positive conversion rate of serum antibody of the vaccine is 88.1–91.7%, and the protective efficacy against EV71 infection associated HFMD is more than 90% [7]. The main vaccination population is children under five years old [18]. We obtained daily vaccination data from Zhejiang CDC, which was officially launched in August 2016 in Zhejiang, including the time of receipt of the two doses and the date of birth of the participants. Considering that the main vaccinated population was children younger than 5 years old, we calculated the cumulative daily vaccine coverage of children under 5 years old based on yearly demographic data and daily vaccination data [19]. The following equation was utilized to calculate the vaccination coverage rate:

Daily vaccination coverage rate = Daily vaccinated number under 5 years of age/number of people under 5 years of age of that year.

### 2.3. Meteorological Data

We extracted the 24 h surface temperatures and relative humidity with a resolution of 0.1° × 0.1° from ERA5-Land (European Centre for Medium-Range Weather Forecasts Reanalysis 5 for Land Surface) reanalysis data to estimate the daily average temperature and humidity [20]. Subsequently, we used the bilinear interpolation approach to estimate the daily meteorological measures for each HFMD case and then averaged the data.

### 2.4. Effective Reproduction Number and Epidemic Model Analysis

The basic reproduction number (*R*_0_) is the expected number of secondary cases that each infected primary case would infect in an entirely susceptible population without any intervention. Furthermore, *R_t_* is a key variable for characterizing pathogen transmissibility during an epidemic, and, compared to case numbers, it offers more direct evidence of HFMD epidemics and the impact of vaccines on its transmission, with an *R_t_* value less than 1 indicating that the epidemic is under control [21,22].

To estimate the *R_t_*, we defined the epidemic duration for different types of HFMD. Specifically, we first defined the HFMD epidemic during the study period employing a spline-smoothed trajectory of weekly reported case incidences [10]. A continuous increase of not less than 5 weeks was regarded as the beginning of the epidemic, while a continuous decrease lasting at least 8 weeks until the next increase appeared was regarded as its end [11].

Based on each epidemic duration and daily cases of EV71, CA16 and other HFMD types, the branching process model was performed to evaluate the daily *R_t_* for each HFMD serotype. To be specific, we assumed that the serial interval of HFMD followed a shifted Gamma distributed with shift 1, with a mean interval of 3.7 days, an interval standard deviation of 2.6 days, and a size of smoothing time window fixed at 14 days, which were determined by clinical studies conducted in previous years on HFMD [23].

Ideally, the transmission rate without intervention would be influenced by susceptible proportion of the population at time t (*S_t_*) and *R*_0_ [24,25]:

(1) $$R_{t}=R_{0}S_{t}$$

The transmissibility of HFMD (*R_t_*) could be affected by multiple interventions, such as social control measures, meteorological conditions, and susceptible individuals [11]. Therefore, we established the following equation (Equation (2)) [11] and utilized the general additive model (GAM) with log as link function and quasi-Poisson as distribution to fit the associations between factors and *R_t_* (Equation (3)):

(2) $$R_{t}=R_{0}S_{0}e^{\beta_{v}V_{t}+\beta_{v}H_{t}+\beta_{voc}voc+s\left(Rh,{df}_{1}\right)+\beta_{dow}dow+s\left(Temp,\;{df}_{2}\right)+s\left(year,\;{df}_{3}\right)}$$

(3) $$ln(R_{t})={{ln(R}_{0}S_{0})+\beta_{h}H_{t}+\beta }_{v}V_{t}+\beta_{voc}{voc}_{t}+s\left({Rh}_{t},{df}_{1}\right)+\beta_{dow}{dow}_{t}+s\left({Temp}_{t},\;{df}_{2}\right)+s\left({year}_{t},\;{df}_{3}\right)$$

where *ln*(*R*_0_*S*_0_) represents the intercept. *V_t_* is the vaccination number at time t. *H_t_* denotes the daily cumulative incidence of EV17, CA16, or other type of HFMD at time t. *dow_t_* and *voc_t_* represent whether time t is weekday and vocation (including legal holidays and summer and winter vacations). Furthermore, since the effects of meteorological factors are often non-linear, the penalty smoothing spline function (*s*) is used to fit time trend (year), temperature (*Temp*), and relative humidity (*Rh*). The degree of freedom (*df*_1_) for *Time* was chosen as 3, while *df*_2_ and *df*_3_ for temperature and relative humidity were set as 3 [26].

Additionally, based on the peak values of EV71 *R_t_* during each epidemic period and the effectiveness of the EV71 vaccine, we conducted further estimations to determine the level of vaccinated number or coverage rate required to effectively prevent the epidemic of EV71-associated HFMD (Equation (4)). Furthermore, we estimated the relative reduction by age group (0–3, 3–5 years), urban or rural distribution, and GDP level of cities. The definition of GDP level is divided by the GDP of various cities in Zhejiang Province in the 2019 Statistical Yearbook, with Hangzhou, Ningbo, and Wenzhou as high GDP cities, Shaoxing, Jiaxing, Lishui, and Jinhua as median, and Quzhou, Taizhou, Huzhou, and Zhoushan as low.

(4) $$\begin{array}{c} Required\;vaccination\;number={log}_{{(e}^{\beta_{v}})}\frac{1}{{max(R}_{t})} \end{array}$$

### 2.5. Sensitivity Analysis

We conducted sensitivity analyses to test the robustness of the findings. First, we used the time of the second vaccination dose as the standard to calculate the number of vaccinated people and the coverage rate. Second, we considered the effectiveness period of the EV71 vaccine [27], categorizing individuals who have been vaccinated for more than three years as a potentially susceptible group. Thirdly, we applied the 6–71 months as study object to calculated the vaccine effect and epidemic prevention threshold [7].

All analyses were performed in the R Programming Language (version: 4.2.1) based on R packages “*mgcv*” and “*EpiEstim*”.

## 3. Results

### 3.1. Descriptive Results

This study included 473,330 cases of HFMD in Zhejiang Province from August 2016 to December 2019. The epidemic of EV71-associated HFMD exhibited a distinct seasonal and biannual pattern, with a peak season during the spring–summer period and a low season during the summer–autumn period. Among the overall cases, an estimated 56,700 were associated with EV71, 93,152 were attributable to CA16, and 323,478 were connected to other enteroviruses. In addition, vaccine coverage increased slowly until 2017, followed by a steady and sustained upward trend from 2017 to 2019, with a slight decrease in slope from September 2017 to February 2018 (Figure S1). As of 31 December 2019, the cumulative vaccination of EV71 vaccine in Zhejiang Province reached 1,465,351, with a vaccine coverage of 43.63% among children younger than 5 years.

### 3.2. Epidemic Waves and R_t_ of HFMD

**Figure 1:**
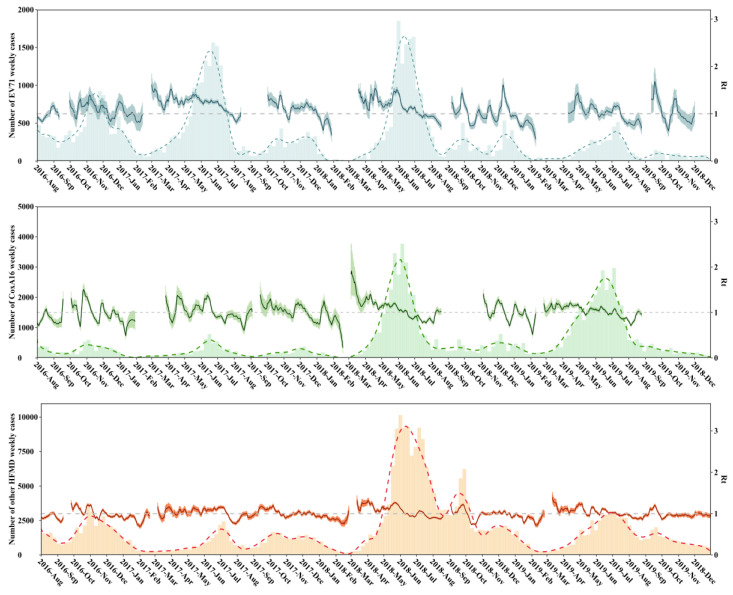
Epidemic period, time-varying effective reproduction number (*R_t_*), and the number of weekly cases and smoothed cases for each serotype of HFMD. EV71 is in blue, CA16 is in green, and other types of HFMD are in orange. The colored bar graphs denote the number of weekly cases, and dashed lines represent the number of smoothed cases. The *R_t_* are represented by the dark curve and existed in the corresponding epidemic period.

During this study period, seven complete epidemic waves of EV71, six waves for CA16, and fives waves for other HFMD types were defined (Figure 1 and Figures S2–S4). Given the bi-peak distribution of the EV71 cases in Zhejiang Province each year, we compared the spring–summer peaks (a) and summer–autumn peaks (b) separately. Specifically, we estimated the median and maximum *R_t_* of EV71, as well as the cumulative incidence of different types of HFMD during each EV71 epidemic (Table 1). We observed that the incidence of EV71-associated HFMD during the summer–autumn peak (2016b, 2017b, 2018b, and 2019b) demonstrated a consistent annual decline. Additionally, there was also a gradual reduction in *R_t_* during the spring–summer peak of EV71 cases. However, for CA16 and other types of HFMD, we observed a decline in the year 2017 followed by a significant increase in 2018, but no consistent decrease was found.

We further estimated the *R_t_* of EV71 before vaccination (2013 to 2016) to demonstrate the transmissibility trend of EV71 before and after vaccination (Table S1). Specifically, for the spring–summer peaks without EV71 vaccination, the *R_t_* was 1.029, 1.011, 1.031, and 1.046 during 2013–2016, which was higher than that in 2017–2019 (0.94, 1.00, and 0.95). While, the *R_t_* of summer–autumn demonstrated a trend of first rising (2013–2016) and then declining (2017–2019).

### 3.3. The Effectiveness of EV71 Vaccination on Transmissibility of HFMD

We observed an inverse association between the EV71 vaccination number and EV71 transmissibility (Table 2). Specifically, we estimated that for every 100,000 children younger than 5 years vaccinated, there was a significant reduction of 14.44% (95%CI: 6.76%, 21.42%) in the *R_t_* of EV71-associated HFMD. We also estimated that each 1% increase in vaccination coverage among children under the age of 5 was associated with a decrease of 5.07% (95%CI: 2.18%, 7.87%) in EV71 *R_t_*. Considering that, as of 2020, the highest peak value of EV71 cases during the seven epidemic periods was 1.76 (Table 1), we assessed that an additional 362,381 vaccinations among children under the age of 5 would be required to prevent outbreaks of EV71, corresponding to an increase in vaccine coverage to 54.51%.

In addition, our study indicated that vaccination does not impact the transmission capacity of CA16, while as for other HFMD types, it might enhance the transmissibility. For instance, vaccinating every 100,000 or increasing 1% vaccination coverage among children under the age of 5 could lead to an increase of su.82% (95%CI: 0.80%, 2.84%) and 0.70% (95%CI: 0.40%, 1.01%) in the *R_t_* of other HFMD types respectively.

### 3.4. The Effectiveness of EV71 Vaccination among Different Age Groups

Compared to children aged 3–5 years old, we found that the preventive effect of vaccination on EV71 transmissibility was stronger when vaccinated those ≤ 3 years old (Table 3). For instance, we found every 100,000 vaccinations of children ≤ 3 years could reduce *R_t_* of 15.21% (95%CI: 6.29%, 23.36%), which was twice as much as those 3–5 years old. Moreover, we also found positive correlations between the vaccinations among children ≤ 3 years old (per 100,000) and the transmissibility of both CA16 (26.11%, 95%CI: 16.42% to 36.62%) and other HFMD types (1.61%, 95%CI: 0.30% to 2.94%), respectively. While no significant associations were observed between the vaccination number of 3–5 years old children and other types of HFMD.

For the stratification analysis by urban or rural distribution (Table S2), we found significant positive associations between vaccination and the *R_t_* of EV71 in both settings. Notably, urban vaccination had a greater impact on EV71 transmissibility (−15.21%, 95%CI: −23.36% to −6.29%) than rural vaccination (−11.26%, 95%CI: −17.11% to −5%). Additionally, we observed significant negative associations between vaccination and other types of HFMD in both rural and urban areas. Furthermore, the stratification findings of GDP (Table S3) demonstrated every 50,000 vaccinations of children <5 in low GDP cities could reduce *R_t_* of 17.08% (95%CI: 8.11%, 25.16%), which was greater than vaccination in high (6.6%, 95%CI: 2.98% to 10.08%) and median GDP (10.19%, 95%CI: 4.18% to 15.82%).

### 3.5. The Results of Sensitivity Analysis

Sensitivity analyses indicated that our results remained robust across different conditions. Firstly, the adjustment that used the second vaccination dose to define the vaccinated number and coverage rate did not significantly change our main results (Table S4). Secondly, the results of the sensitivity analyses that took into account the five-year validity of the vaccine were also similar to our main findings (Table S5). Thirdly, the prevention threshold (an additional 362,381 vaccinations, corresponding to an increase in vaccine coverage to 60.94%) and the association of “vaccine-EV71 transmissibility” that applied 6–71-month-old children as study objects were similar to that of our main analysis (Table S6).

## 4. Discussion

Given the dearth of research on the effectiveness of the EV71 vaccination on the transmissibility of different types of HFMD, we sought to fill this gap and provide more direct evidence for the government to use in preventing HFMD epidemics. We observed that a higher EV71 vaccination number was associated with a lower transmissibility of EV71-associated HFMD, whereas it was higher for other types of HFMD. Our study further estimated the additional vaccination numbers or vaccine coverage that was required to prevent EV71 epidemics. Additionally, our findings also demonstrated the differences in the effectiveness of vaccination in controlling the epidemic of EV71 among different age groups.

Aligned with previous studies, we observed a consistent annual decline of EV71-associated HFMD cases after the vaccination program was carried out, as well as a gradual decrease in EV71 transmissibility during the spring–summer peak of 2017–2019. For instance, Wu et al. found a reduction trend in HFMD cases in Zhejiang Province from August 2016 to December 2019 [12]. Similarly, several longitudinal studies in other regions of China also observed significant reductions in EV71-associated HFMD cases after the initiation of the vaccination [28]. This observation added evidence on the effectiveness of the EV71 vaccination.

We also observed an inverse association between EV71 vaccination and EV71 transmissibility and further assessed the prevention threshold (an additional 362,381 vaccinations among children <5), which proved the effectiveness of EV71 vaccination and set a specific objective for vaccination program. However, EV71, as a self-funded, second-class vaccine in China, was often affected by the family income and individual’s awareness of vaccination importance [29]. Therefore, the government should pay more attention to the popularization of EV71 virus knowledge and its serious health consequences, rather than just the vaccine itself [29].

Moreover, the evidence of the epidemic period *R_t_* calculated when the EV71 vaccine was not available in Zhejiang Province (Table S1) also suggested that the EV71 vaccination could reduce the transmissibility of EV71-associated HFMD. For instance, both the reduction trend of *R_t_* in 2013–2019 spring–summer period and the consistent annual decline from 2017 to 2019 demonstrated the impact of EV71 vaccination on EV71 transmissibility. Meanwhile, the high levels of *R_t_* in summer–autumn 2017 could be explained by the short-term fluctuations of EV71 when the vaccine had not reached high levels.

Notably, our results demonstrated that a higher EV71 vaccination number was associated with a strong transmission of CA16 and other types of HFMD, which was also aligned with previous findings [30]. This feature could be explained by the potential competitive interaction among different types of HFMD [31]. Specifically, when the EV71 vaccine reduces the transmission of the EV71 virus, it may create an ecological opportunity for other HFMD-causing viruses, like CA16 or other types, to spread more easily. Moreover, the observation added evidence that EV71 vaccination had no protection against CA16 or other types of HFMD, suggesting that the combined effect of the vaccine on EV71 and non-EV71 enteroviruses should not be ignored in the HFMD prevention and control [6].

For the distribution-stratified findings, we observed that vaccination programs were more effective at controlling HFMD transmission in urban populations compared to rural areas. Several mechanisms could explain this difference. Given the rapid urbanization of Zhejiang in recent years, many children, the primary patients of HFMD, relocated to cities for better educational opportunities. As a result, rural schools experienced a decline in student number, while kindergartens and primary schools, as the major targets of HFMD epidemic and mainly situated in urban areas [32], experienced an increased trend of student number. In these institutions, where susceptible populations were more densely concentrated and the virus spread more easily [33], vaccination proved to be more effective in preventing HFMD transmission. Additionally, our GDP-stratified results suggested that vaccination in low-GDP cities could better control the transmissibility of EV71. Cities with lower GDP often experienced higher population density and poorer sanitation than rural areas, both of which were critical risk factors for HFMD transmission [32,34]. Therefore, vaccinating in regions with a higher risk of HFMD could more effectively control EV71 transmission.

Furthermore, combining our previous findings with age-stratified results in this study, we could conclude that the EV71 vaccination program could prevent a greater percentage of EV71 cases among children aged 3–5 years [19], while vaccination in children under 3 years old could better control the transmissibility of EV71. This difference suggested that, even though the vaccine program prevented a greater percentage of children aged 3–5 years [19], it was the children under 3 years of age that might be the primary infectious population for EV71 transmission. Due to the weak immunity and poor hygiene habits of scattered children (in China, children under age 3 are usually scattered) [35], they might transmit the enterovirus to nursery children, leading outbreaks in kindergartens and widespread epidemics. Thus, in terms of case number, the kindergarten children demonstrated a higher case number and a greater preventive percentage due to the EV71 vaccination [19]. These findings indicated that strengthening the vaccination of children under 3 years old could be more effective in controlling EV71 transmissibility and preventing further epidemics in future vaccination strategy.

There were several limitations in this study. First, as this study was based on just three years (2017–2019), our results may be influenced by short-term fluctuations in EV71 cases and could not reveal vaccine effectiveness after the COVID-19 period, during which the pandemic might change the epidemic feature of HFMD. Therefore, future studies should consider longer-term cohort data or more diverse datasets after the COVID-19 epidemic to provide a more comprehensive evaluation of the vaccination. Second, the association of vaccination with HFMD transmissibility might be affected by other factors as our design was not able to consider all of the factors. Thirdly, our model assumed no cross-immunity between different enteroviruses, which might lead to an underestimation of the immunized people and further influence the assessment of vaccine effectiveness. Finally, the study findings might not be generalizable to regions with different climates, healthcare systems, or vaccination policies. However, the method for evaluating vaccine effectiveness and its epidemic prevention threshold could be widely applicable.

## 5. Conclusions

In conclusion, this study observes that EV71 vaccination could effectively reduce the transmissibility of EV71-associated HFMD and slightly increase the transmissibility of non-EV71-HFMD. This indicates the necessity for enhancing EV71 vaccination coverage, alongside addressing non-EV71 HFMD, and advancing the development of a multi-valent HFMD vaccine. Additionally, our findings underscore the efficacy of targeting children under 3 years old in controlling EV71 transmission and further outline the EV71 vaccination thresholds for the government to promote as the vaccination program.

## Figures and Tables

**Table 1:** The median and maximum *R_t_* of EV71 and cumulative incidence of different types of HFMD, as well as the vaccination number and vaccination rate of children ≤ 5 years during each EV71 epidemic.

Epidemic Period of EV71	The Median of EV71 *R_t_*	The Maximum of EV71 *R_t_*	Cumulative Incidence of EV71	Cumulative Incidence of CA16	Cumulative Incidence of Other Type	Vaccination Number of Children ≤ 5 Years (Vaccination Rate)
2016b	1.055	1.29	9860	5012	35,641	2075 (0.07%)
2017a	0.94	1.41	15,214	5733	19,925	50,807 (1.71%)
2017b	1.05	1.30	4104	3331	22,049	303,612 (10.23%)
2018a	1.00	1.76	14,615	30,743	115,891	443,188 (14.56%)
2018b	1.03	1.61	4667	8430	55,679	809,976 (26.61%)
2019a	0.95	1.44	4211	32,681	41,931	1,094,957 (32.61%)
2019b	1.01	1.68	992	3452	16,454	1,337,906 (39.84%)

**Table 2:** Associations of vaccination number, vaccination rate, and *R_t_* of different types of HFMD.

Type	Vaccination Number (per 100,000 Children ≤ 5 Years)	Vaccination Rate (1%)
EV71	−14.44% (−21.42%, −6.76%)	−5.07% (−7.87%, −2.18%)
CA16	0.40% (−0.90%, 1.82%)	0.30% (−0.10%, 0.70%)
Others	1.82% (0.80%, 2.84%)	0.70% (0.40%, 1.01%)

Note: The values in the table represent the percentage decrease in EV71 *R_t_* for every additional 100,000 people vaccinated. For example, the “−14.44% (−21.42%, −6.76%)” represents a decrease of 16.5% in EV71 *R_t_* for every 100,000 people vaccinated.

**Table 3:** The associations between vaccination number and *R_t_* of each type of HFMD among different age groups.

Type	EV71	CA16	Others
Vaccination number (per 100,000 children ≤ 3 years)	−15.21% (−23.36%, −6.29%)	26.11% (16.42%, 36.62%)	1.61% (0.30%, 2.94%)
Vaccination number (per 100,000 children 3–5 years)	−8.42% (−14.36%, −2.08%)	1.51% (−0.30%, 3.25%)	−0.90% (−2.37%, 0.60%)

Note: The values in the table represent the percentage decrease in EV71 *R_t_* for every additional 100,000 people vaccinated, for example. For example, the “−15.21% (−23.36%, −6.29%)” represents a decrease of 16.5% in EV71 *R_t_* for every 100,000 people vaccinated.

## Data Availability

The data supporting the findings of this study are sourced from National Notifiable Infectious Diseases Reporting Information System (NNIDRIS) containing sensitive information. Due to privacy restrictions, the data cannot be made publicly available. However, specific data could be shared with researchers upon reasonable request from correspondence authors.

## Supplementary Materials

The following supporting information can be downloaded at https://www.mdpi.com/article/10.3390/vaccines12101166/s1. Figure S1: The increase pattern of EV71 vaccine coverage in Zhejiang Province during 2016 to 2019. Figure S2: The growth rate of the estimated daily number of other type of HFMD per week. Figure S3: The growth rate of the estimated daily number of CA16-associated HFMD per week. Figure S4: The growth rate of the estimated daily number of EV71-associarted HFMD per week; Table S1: The epidemic period of EV71 and the median of EV71 *R_t_* for each epidemic period. Table S2: The associations between urban and rural vaccination number and *R_t_* of each HFMD type. Table S3: The associations between urban and rural vaccination number and *R_t_* of each HFMD type. Table S4: The association between vaccination number and *R_t_* of each type of HFMD after regarding the date of the second vaccination dose as the standard to calculate vaccination number. Table S5: The association between vaccination number and *R_t_* of each type of HFMD after considering those who have been vaccinated for more than three years as susceptible individuals. Table S6: Associations of vaccination number, vaccination rate and Rt of different types of HFMD after applied the 6–71 months as study object.
