# Supplementary material for: Effect of EV71 Vaccination on Transmission Dynamics of Hand, Foot, and Mouth Disease and Its Epidemic Prevention Threshold
Source: Vaccines (Basel). 2024 Oct 12;12(10):1166. doi: 10.3390/vaccines12101166 (PMC11511198; doi:10.3390/vaccines12101166)
Supplement: Supplementary file 1 [file vaccines-12-01166-s001.zip › vaccines-3231131-supplementary.pdf]

<u>SUPPLEMENTAL MATERIALS</u>

Table S1. The epidemic period of EV71 and the median of EV71 R_t for each epidemic period.

Epidemic period of EV71	The median of EV71 R_t	Begin timing of epidemic period
2013a	1.029	2013-02
2013b	0.980	2013-08
2014a	1.011	2014-02
2014b	0.936	2014-08
2015a	1.031	2015-03
2015b	0.994	2015-08
2016a	1.046	2016-02

Table S2. The associations between urban and rural vaccination number and Rt of each HFMD type.

Type	EV71	CA16	Others
Vaccination number (per 50,000 children in urban)	-15.88% (-23.8%, -7.14%)	0.04% (-1.65%, 1.76%)	1.44% (0.24%, 2.66%)
Vaccination number (per 50,000 children in rural)	-11.26% (-17.11%, -5%)	0.33% (-0.76%, 1.43%)	1.12% (0.35%, 1.89%)

Note: The values in the table represent the percentage decrease in EV71 R_t for every additional 50,000 people vaccinated, for example. For example, the '-15.88%' represents a decrease of 16.5% in EV71 R_t for every50,000 people vaccinated.

Table S3. The associations between urban and rural vaccination number and R_t of each HFMD type.

Type	EV71	CA16	Others
Vaccination number (per 25,000 children in High GDP regions)	-6.6% (-10.08%, -2.98%)	-0.07% (-0.73%, 0.6%)	0.54% (0.06%, 1.03%)
Vaccination number (per 25,000 children in Median GDP regions)	-10.19% (-15.82%, -4.18%)	0.55% (-0.42%, 1.54%)	0.97% (0.33%, 1.62%)
Vaccination number (per 25,000 children in Low GDP regions)	-17.08% (-25.16%, -8.11%)	0.51% (-1.12%, 2.17%)	1.98% (0.75%, 3.22%)

Note: The values in the table represent the percentage decrease in EV71 R_t for every additional 25,000 people vaccinated, for example. For example, the '-6.6%' represents a decrease of 6.6% in EV71 R_t for every 25,000 people vaccinated.

Table S4. The association between vaccination number and R_t of each type of HFMD after regarding the date of the second vaccination dose as the standard to calculate vaccination number.

Type	EV71	CA16	Others
Vaccination number (per 100,000 children ≤5 years)	-12.98% (-20.31%, -4.97%)	0.1% (-1.29%, 1.49%)	1.31% (0.2%, 2.33%)
Vaccination number (per 100,000 children ≤3 years)	-9.06% (-15.46%, -2.08%)	26.24% (15.72%, 37.58%)	0.7% (-0.7%, 2.22%)
Vaccination number (per 100,000 children 3-5 years)	-15.8% (-24.65%, -5.92%)	1.31% (-0.7%, 3.36%)	-1.29% (-2.76%, 0.2%)

Table S5. The association between vaccination number and R_t of each type of HFMD after considering those who have been vaccinated for more than three years as susceptible individuals.

Type	EV71	CA16	Others
Vaccination number (per 100,000 children ≤ 5 years)	-14.62% (-21.65%, -6.85%)	0.40% (-0.90%, 1.82%)	1.92% (0.90%, 2.94%)
Vaccination number (per 100,000 children ≤ 3 years)	-29.74% (-40.25%, -17.39%)	26.11% (16.42%, 36.62%)	1.71% (0.40%, 3.05%)
Vaccination number (per 100,000 children 3-5 years)	-17.14% (-28.18, -4.50%)	1.51% (-0.30%, 3.25%)	-0.90% (-2.37%, 0.6%)

Table S6. Associations of vaccination number, vaccination rate and R_t of different types of HFMD after applied the 6-71 months as study object.

Type	Vaccination number (per 100,000 children 6-71 months)	Vaccination rate (1%)
EV71	-13.02% (-19.62%, -5.89%)	-7.10% (-11.69%, -2.27%)
CA16	0.24% (-1.53%, 1.05%)	0.14% (-0.27%, 0.56%)
Other	1.14% (0.25%, 2.04%)	0.50% (0.21%, 0.79%)

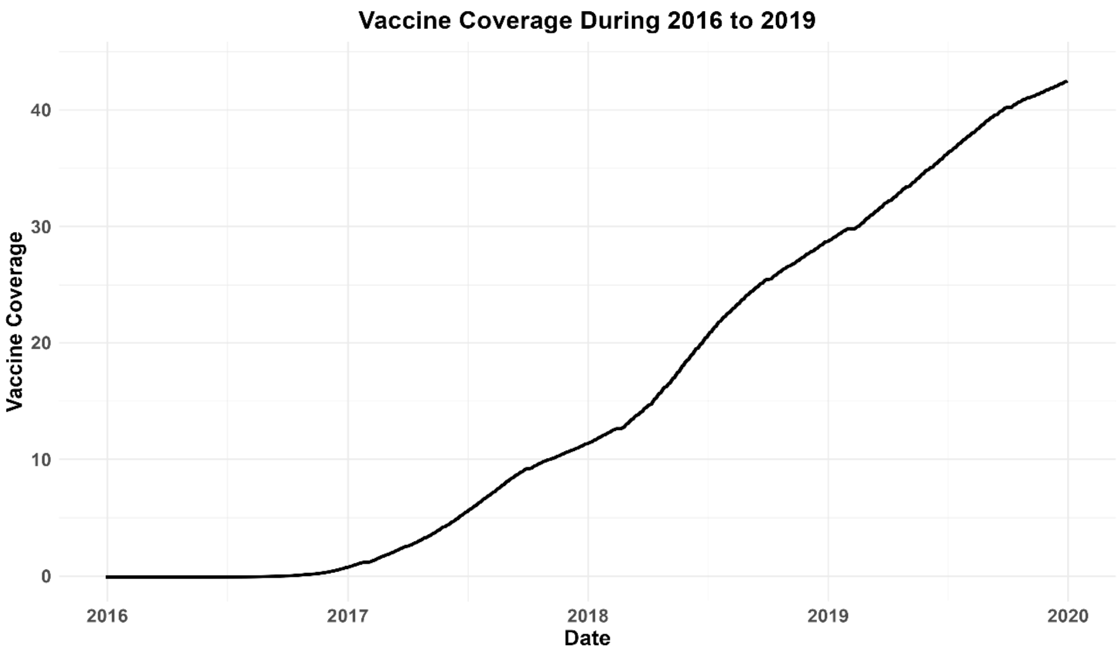

Figure S1. The increase pattern of EV71 vaccine coverage in Zhejiang Province during 2016 to 2019.

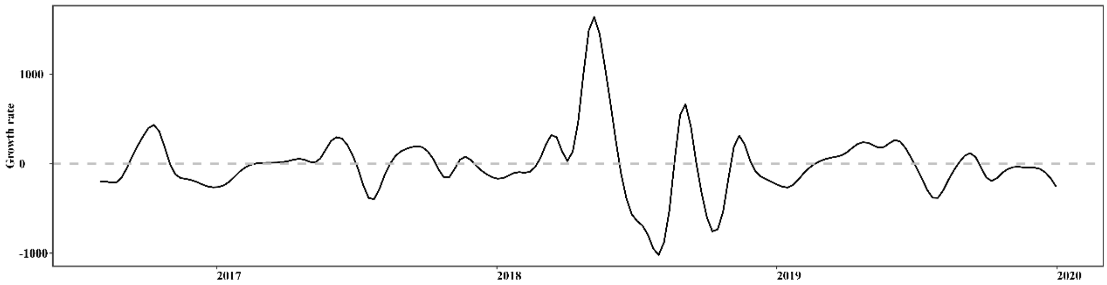

Figure S2 The growth rate of the estimated daily number of other type of HFMD per week.

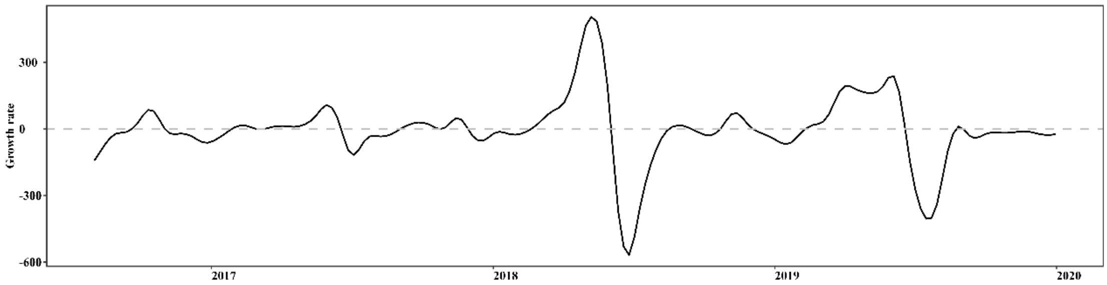

Figure S3 The growth rate of the estimated daily number of CA16-associated HFMD per week.

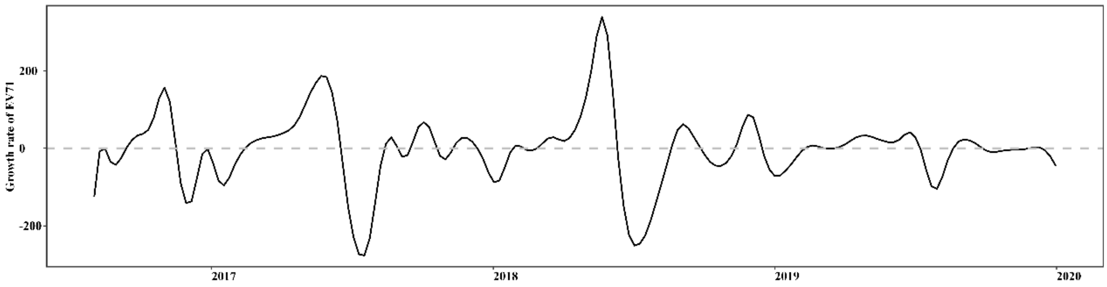

Figure S4 The growth rate of the estimated daily number of EV71-associarted HFMD per week.

The process of calculability of the equation (4):

For the log-linear transformation, $ln(Y_i) = \mathrm{a} + bX_i$, b can be interpreted as: for each unit increase in vaccination, the average estimated change in $ln(Y_i)$.

When X takes the value X_1, $ln(Y_1) = \mathrm{a} + bX_1$; when X takes the value X_2, $ln(Y_2) = \mathrm{a} + bX_2$. Thus, assuming that when X increases by one unit, $ln(Y_2) - ln(Y_1) = (a + bX_1) - (\mathrm{a} + bX_2) = b$, we get: $b = ln(Y_2) - ln(Y_1) = ln\frac{Y_2}{Y_1}$, thus, $\frac{Y_2}{Y_1} = e^b$, then $Y_2 = Y_1e^b$.

The relationship between $Y_2$and Y_1 can be expressed as a percentage change:

$$\frac{Y_{2-}Y_1}{Y_1} \times 100\% = \frac{Y_1e^b - Y_1}{Y_1} \times 100\% = (e^b - 1) \times 100\%$$

Thus, after X changes by 1 unit, Y becomes:

$$[1 + (e^b - 1) \times 100\%] \times Y$$

Therefore, in our study, if we denote that the independent variable X needs to increase by *d* units for Y to change from $\max(R_t)$ to 1, it can be represented using the following formula:

$$\max(R_t)\,[1 + (e^{\beta_v} - 1) \times 100\%]^d = 1$$

Notably, in this study *d* represents required vaccination number, which could be estimated by formula (4):

$$ln(e^{\beta_v})^{Required\ vaccination\ number} = ln\,(\frac{1}{\max\,(R_t)})$$

thus,

$$Required\ vaccination\ number = log_{(e^{\beta_v})}\frac{1}{\max(R_t)}$$
